# Up‐regulated deubiquitinase USP4 plays an oncogenic role in melanoma

**DOI:** 10.1111/jcmm.13603

**Published:** 2018-03-14

**Authors:** Weinan Guo, Jinyuan Ma, Tianli Pei, Tao Zhao, Sen Guo, Xiuli Yi, Yu Liu, Shiyu Wang, Guannan Zhu, Zhe Jian, Tianwen Gao, Chunying Li, Wenjun Liao, Qiong Shi

**Affiliations:** ^1^ Department of Dermatology Xijing hospital Fourth Military Medical University Xi'an Shaanxi China

**Keywords:** melanoma, metastasis, p53, ubiquitination, USP4

## Abstract

Melanoma is the most malignant skin cancer with increasing incidence worldwide. Although innovative therapies such as BRAF inhibitor and immune checkpoint inhibitor have gained remarkable advances, metastatic melanoma remains an incurable disease for its notorious aggressiveness. Therefore, further clarification of the underlying mechanism of melanoma pathogenesis is critical for the improvement of melanoma therapy. Ubiquitination is an important regulatory event for cancer hallmarks and melanoma development, and the deubiquitinating enzymes including ubiquitin‐specific peptidase (USP) families are greatly implicated in modulating cancer biology. Herein, we first found that the expression of the deubiquitinase USP4 was significantly up‐regulated in melanoma tissues and cell lines. Furthermore, although USP4 knockdown had little impact on melanoma cell proliferation, it could increase the sensitivity to DNA damage agent cisplatin. We subsequently showed that USP4 regulated cisplatin‐induced cell apoptosis via p53 signalling. More importantly, USP4 could accentuate the invasive and migratory capacity of melanoma cells by promoting epithelial‐mesenchymal transition. Altogether, our results demonstrate that the up‐regulated USP4 plays an oncogenic role in melanoma by simultaneously suppressing stress‐induced cell apoptosis and facilitating tumour metastasis.

## 1 INTRODUCTION

Melanoma is the most lethal skin cancer originated from the malignant transformation of melanocyte.^1^ In 2016, there are estimated 87 000 new cases of melanoma in the USA and over 9700 deaths from it.^2^ Recently, some innovative therapies including BRAF/MEK inhibitors and immune checkpoint inhibitors have gained revolutionary advances for the outcome of melanoma patients.^1^ However, for the most part, the metastatic melanoma remains an incurable disease for its notorious aggressiveness, and the survival rate can be even <20% upon the occurrence of metastasis.^3^, ^4^, ^5^ Therefore, to further clarify the mechanism underlying melanoma pathogenesis is of great importance for improving the efficiency of melanoma therapy and the management of melanoma patients.

Ubiquitination is one of the most important post‐translational modifications reversibly regulated by both ubiquitin‐conjugating and ubiquitin‐deconjugating enzymes.^6^ Depending on the specificity of ubiquitinated substrates and linkage types, ubiquitination participates in a wide range of processes, including metabolism, cell proliferation, inflammatory response and autophagy,^7^, ^8^, ^9^, ^10^ which are highly associated with cancer hallmarks. For melanoma, the mutations of ubiquitination‐related genes such as *BRCA1‐associated protein‐1(BAP1), F‐boxand WD repeat domain‐containing 7 (FBXW7) and PARK2* are frequently identified and participated in melanoma tumourigenesis.^11^ What is more, the activation of some critical signalling pathways in melanoma development including NF‐κB and MITF are also subtly regulated by ubiquitination,^12^, ^13^ demonstrating its vital pathogenic role in melanoma. Notably, previous studies mainly focused on the regulatory role of ubiquitin‐conjugating enzymes, especially E3 ubiquitin ligases in melanoma. However, the effect of deubiquitinating enzymes (DUBs), which mediate the removal and processing of ubiquitin, remains far from understood.

Ubiquitin‐specific proteases (USPs) represent the largest family of DUBs with over 60 members in humans.^14^ Among them, USP4 is a rather important USP member with previously reported links to cancer associated‐DNA repair and p53 stability.^15^, ^16^, ^17^, ^18^ On the one hand, the elevated USP4 expression contributed to the growth of colorectal cancer via deubiquitination and stabilization of oncogene PRL‐3.^19^ On the other hand, USP4 could also exert its tumour‐suppressive role through inhibiting cell proliferation or promoting cell apoptosis in breast cancer and neck squamous cell carcinoma.^20^, ^21^ These results suggested that the impact of USP4 on cancer biology is tumour‐type specific and tissue‐context dependent. Nevertheless, whether USP4 is aberrantly expressed and plays a pathogenic role in melanoma have not been investigated.

In the present study, we first found that the expression of USP4 was remarkably up‐regulated in both melanoma cell lines and tissues, and USP4 expression was higher in metastatic melanoma than that in primary one. Subsequently, we showed that the knockdown of USP4 had little effect on melanoma cell proliferation and colony formation under normal condition. However, in response to cisplatin treatment, USP4 deficiency significantly augmented melanoma cells apoptosis by activating p53 signalling. Moreover, we found that USP4 was also able to potentiate the invasive and migratory capacity of melanoma cells by promoting epithelial–mesenchymal transition. Altogether, our results demonstrate that the up‐regulated USP4 plays an oncogenic role in melanoma by simultaneously inhibiting stress‐induced cell apoptosis and facilitating tumour metastasis.

## 2 MATERIALS AND METHODS

### 2.1 Clinical specimens

Tissue samples for immunofluorescence analysis were taken from 23 melanoma patients (11 patients at primary stage and 12 patients at metastatic patients) and 10 nevus cases after the histological confirmation. All the clinical specimens were obtained in Department of Dermatology, Xijing Hospital, the Fourth Military Medical University. The research protocol was designed and executed according to the principles of the Declaration of Helsinki and was approved by the ethics review board of Fourth Military Medical University. Written informed consent was obtained from all patients and cancer‐free controls.

### 2.2 Cell culture and reagents

Normal human melanocyte cell line NHEM was cultured in Ham's F10 media supplemented with ITS premix (Becton Dickinson, Franklin Lakes, NJ). Human melanoma cell lines WM793B, WM35, A2058, A375 and 451Lu were obtained from American Type Culture Collection (ATCC, Manassas, VA). WM35 and 451Lu cell lines were cultured in RPMI 1640 medium (Invitrogen) supplemented with 10% foetal bovine serum (Invitrogen). WM793B cell line was maintained in MCDB153 medium with 10% foetal bovine serum (Invitrogen). A2058 and A375 cell lines were cultured in Dulbecco's MEMV F12 medium (Invitrogen) supplemented with 10% foetal bovine serum (Invitrogen). Cisplatin (Sigma‐Aldrich, St Louis, MO) was used at a concentration of 25 μmol/L unless specified description.

### 2.3 Total RNA isolation and qRT‐PCR

Total RNA was isolated using TRIzol reagent (Invitrogen) and then reversely transcribed to single‐strand cDNA using reverse transcription reagents (TaKaRa, Dalian, China) according to the manufacturer's instructions. QRT‐PCR experiments were performed using the SYBR Mix (TaKaRa) and Bio‐Rad Multicolor Real‐time PCR Detection System (iQTM5, Bio‐Rad). The cycling conditions were as follows: 95°C for 2 minutes followed by 40 cycles of denaturation at 95°C for 5 seconds, annealing at 55°C for 10 seconds, and extension at 72°C for 15 seconds. All reactions were run in triplicate. The resulting amplification and melt curves were analysed to ensure the identity of the specific PCR product. Threshold cycle values were used to calculate the fold change in the transcript levels by using the 2^−ΔΔCT^ method. The relative mRNA expression levels were normalized to the ACTB gene. The primer sequences are as follows: *USP4*, forward: ACCATTGCAACCATCGAGAA, reverse, TTTTGACTGCAAGGTCTGCC; *P53*, forward: CACGTACTCTCCTCCCCTCA, reverse, CTTCTGTACGGCGGTCTCTC; *ACTB*, forward: AGAAAATCTGGCACCACACC, reverse, AGAGGCGTACAGGGATAGCA;

### 2.4 Protein preparation and immunoblotting

Total protein extracts were obtained using a boiling buffer containing 0.125 mol/L Tris/HCl, pH 6.8 and 2.5% sodium dodecyl sulphate (SDS). In all, 30 μg of proteins was separated by SDS–polyacrylamide gel electrophoresis (PAGE) and electroblotted onto polyvinylidene fluoride membranes (Millipore, Billerica, MA). Protein expression was detected using primary and secondary antibodies and visualized with Image J software for the densitometry analysis of each band.

### 2.5 Antibodies and reagents

Anti‐USP4 antibody (ab181105), anti‐GAPDH antibody (ab8245), anti‐p53 antibody (ab32389), anti‐E‐cadherin antibody (ab15148) and anti‐N‐cadherin antibody (ab98952) were purchased from Abcam, MA; anti‐cleaved‐caspase9 (#9505), anti‐Bcl2 antibody (#2872) and anti‐Bax antibody (#5023) were purchased from Cell Signaling Technology, Inc.

Secondary antibodies were goat anti‐rabbit IgG (1:5000, 111‐035‐003, Jackson ImmunoResearch, WestGrove, PA) and goat anti‐mouse IgG (1:5000, 115‐035‐003, Jackson ImmunoResearch).

### 2.6 Immunofluorescence staining analysis

Immunofluorescence staining was carried out as described previously.^22^ In brief, paraffin‐embedded tissue sections were deparaffinized and rehydrated with graded ethanol dilutions. After antigen retrieval in Tris–EDTA buffer (10 mmol/L Tris base, 1 mmol/L EDTA solution, 0.05% Tween‐20, pH 9.0), immunofluorescence staining was performed by incubating the paraffin sections with a primary rabbit monoclonal anti‐USP4 antibody (1:250, ab181105, Abcam) and a primary mouse monoclonal anti‐MelanA antibody (1:100, ab187369, Abcam) overnight at 4°C, followed by 1‐hour incubation with secondary antibodies Alexa Fluor 488 anti‐rabbit IgG (1:200, #4412, Cell Signaling Technology) and Cy3 anti‐mouse IgG (1:200, ab97035, Abcam) at room temperature. DAPI (Dako, Glostrup, Denmark) was used as a counterstain. Tissue sections were analysed by confocal laser scanning microscopy (FV‐1000, Olympus, Tokyo, Japan). We further used Image J software for the densitometry analysis.

### 2.7 Short hairpin RNA transfection

The human shRNA lentiviral transduction particles targeting USP4 and the control particles were purchased from GenePharma (Shanghai, China). Cells were seeded at approximately 30% confluency. One day later, the medium was aspirated and fresh medium without antibiotics was added. The cells were transduced with human shRNA lentiviral transduction particles for 48 hours. The shRNA sequences were as follows: Sh‐UPS4‐1, 5′‐CCCAACTGTAAGAAGCATCAA‐3′; Sh‐USP4‐2, 5′‐GCCCAGAATGTGCTAAGGTTT‐3′.

### 2.8 Cell proliferation assay

Cell proliferation was monitored using a Cell Counting Kit‐8(CCK‐8) according to the manufacturer's protocol (Beyotime, China). Briefly, melanoma cells were initially seeded at 5 × 10^3^ cells per well in 96‐well plates overnight. Then, CCK‐8 solution was added to each culture well and incubated for 30 minutes at 37°C. Absorbance was read at 450 nm using a Synergy 2 multi‐detection microplate reader.

### 2.9 Colony formation assay

The ability of cells to form colonies was evaluated on a monolayer surface. After melanoma cells were transfected with USP4 shRNA lentiviral vector, adherent cells were trypsinized and counted, and viability was determined. Of those viable cells, 2000 cells were reseeded in a 6‐well plate (in triplicate). Cells were allowed to adhere and grow for between 12 and 14 days. To visualize colonies, cells were fixed in 100% ethanol and stained with crystal violet staining solution (Sigma‐Aldrich). Colonies were counted with an Image J software.

### 2.10 Cell apoptosis analysis

Melanoma cells transfected with USP4 shRNA were seeded in 6‐well plates at a density of 2.5 × 10^5^ cells per well overnight, and the cells were then harvested by trypsinization, washed twice with 4°C PBS, and re‐suspended in binding buffer. Annexin V‐PE and 7AAD solution (NeoBioscience, Shanghai, China) were then added to stain the cells before analysis by flow cytometry (Beckman Coulter, Miami, FL). Each sample was repeated in triplicate.

### 2.11 Invasion and migration assays

#### 2.11.1 Transwell invasion and migration assay

Melanoma cells were transfected with indicated molecules. Thirty‐six hours later, cells were starved in serum‐free medium for additional 12 hours and then trypsinized for reseeding on the top chambers of 24‐well transwell culture inserts (Corning, NY). After 24 hours, cells were fixed in 4% paraformaldehyde for 10 minutes at room temperature. For invasion assays, transwell chambers with 8‐μm pore‐size membrane filter inserts (Corning) coated with Matrigel (BD Biosciences, NJ) were used to determine cell invasion. The non‐motile or non‐invasive cells on the upper side of the filter were removed, while the motile or invasive cells on the lower side were stained with crystal violet. For the quantification of the invasive and migratory cells, we used the “Multi‐point” tool in Image software to count the stained cells. Five fields for each well were counted under the inverted system microscope (Ti‐S, Nikon, Tokyo, Japan).

#### 2.11.2 Wound‐healing assay

Freshly confluent monolayers of melanoma cells transfected with the indicated molecules were wounded by manually scraping off cells with a sterile pipette tip. All the wound sizes were verified to ensure that they were all the same width (approximately 0.8 mm). The cell culture medium was then replaced with serum‐free medium containing 4 μg/mL mitomycin C (Sigma, MO), and wound closure was monitored over a 48‐hour period with a phase contrast microscope at ×200 magnification (Olympus, Tokyo, Japan).

### 2.12 Statistical analyses

Unless otherwise noted, the results were analysed by two‐tailed Student's *t* test, and the results are presented as mean ± SD through at least 3 independent experiments, with *P <* *.05; **P <* *.01; ***P <* *.001 considered to be statistically significant. All statistical analyses were done using SPSS 17.0 (SPSS Inc, Chicago, IL).

## 3 RESULTS

### 3.1 USP4 expression is significantly up‐regulated in melanoma

**Figure 1:**
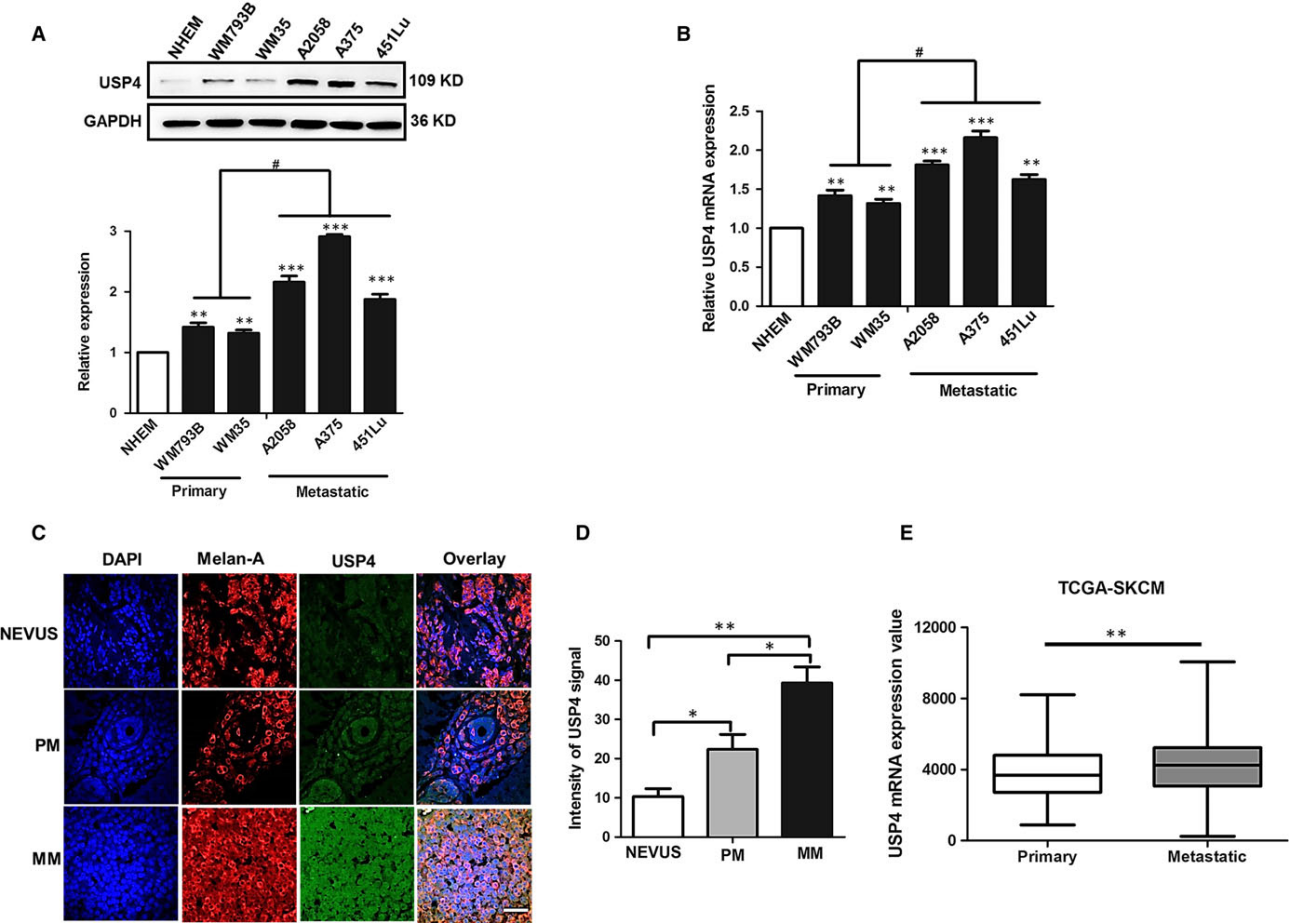
USP4 expression is significantly up‐regulated in melanoma cell lines and tissues. A, USP4 protein expression was analysed by immunoblotting in normal human melanocyte (NHEM) and melanoma cell lines at different stages. The lower panel is a densitometry analysis of 3 individual experiments. B, USP4 mRNA level was analysed by qRT‐PCR in NHEM and melanoma cell lines at different stages. C, Representative immunofluorescence staining images of USP4 expression in nevus and melanoma tissues. Scale bar = 100 μm. D, Densitometry analysis of USP4 signalling intensity in immunofluorescence staining images, related to C. E, USP4 mRNA expression value of melanoma tissues at different stages in TCGA skin cutaneous melanoma (SKCM) database. Data are presented as the mean ± SD, *P <* *.05, **P <* *.01, ***P <* *.001, NHEM and Nevus as control, respectively; ^#^ *P *<* *.05, Primary stage as control; Student's *t* test

To explore the role of USP4 in melanoma, we first examined the mRNA and protein level of USP4 in a panel of melanoma cell lines at different clinical stages (WM793B, WM35 at primary stage and A2058, A375, 451Lu at metastatic stage), as well as in normal human melanocyte (NHEM). Our western blotting analysis showed that the USP4 expression was markedly increased in melanoma cells compared with that in NHEM, though the expression level varied a lot in different melanoma cell lines. Notably, the overall USP4 expression was higher in metastatic melanoma cells than that in primary ones (Figure 1A). In consistent, our real‐time quantitative reverse transcription‐PCR (qRT‐PCR) assay also revealed that the mRNA level of USP4 was prominently up‐regulated in melanoma cell lines, and USP4 expression was slightly higher in metastatic cells than that in primary ones at the transcriptional level (Figure 1B). Furthermore, we performed immunofluorescence staining analysis for detection of USP4 expression in a cohort of 23 melanoma tissues (11 tissues of primary stage and 12 tissues of metastatic stage), as well as in 10 nevus tissues. Our results showed that USP4 expression was nearly undetectable in nevus tissues, whereas it was significantly increased in melanoma tissues (Figure 1C). More importantly, the intensity of USP4 in metastatic melanoma tissues was markedly higher than in primary ones (Figure 1D), which was in line with the results in melanoma cells. What's more, we turned to TCGA skin cutaneous melanoma (SKCM) database to analyse the status of USP4. The mRNA expression of USP4 was significantly higher in metastatic melanoma compared with that in primary melanoma in TCGA database, further confirming our results in both melanoma cell lines and tissues (Figure 1E). Taken together, these results demonstrated that USP4 expression was up‐regulated in melanoma, indicating that USP4 may act as an oncogene.

### 3.2 USP4 is dispensable for melanoma cell proliferation

**Figure 2:**
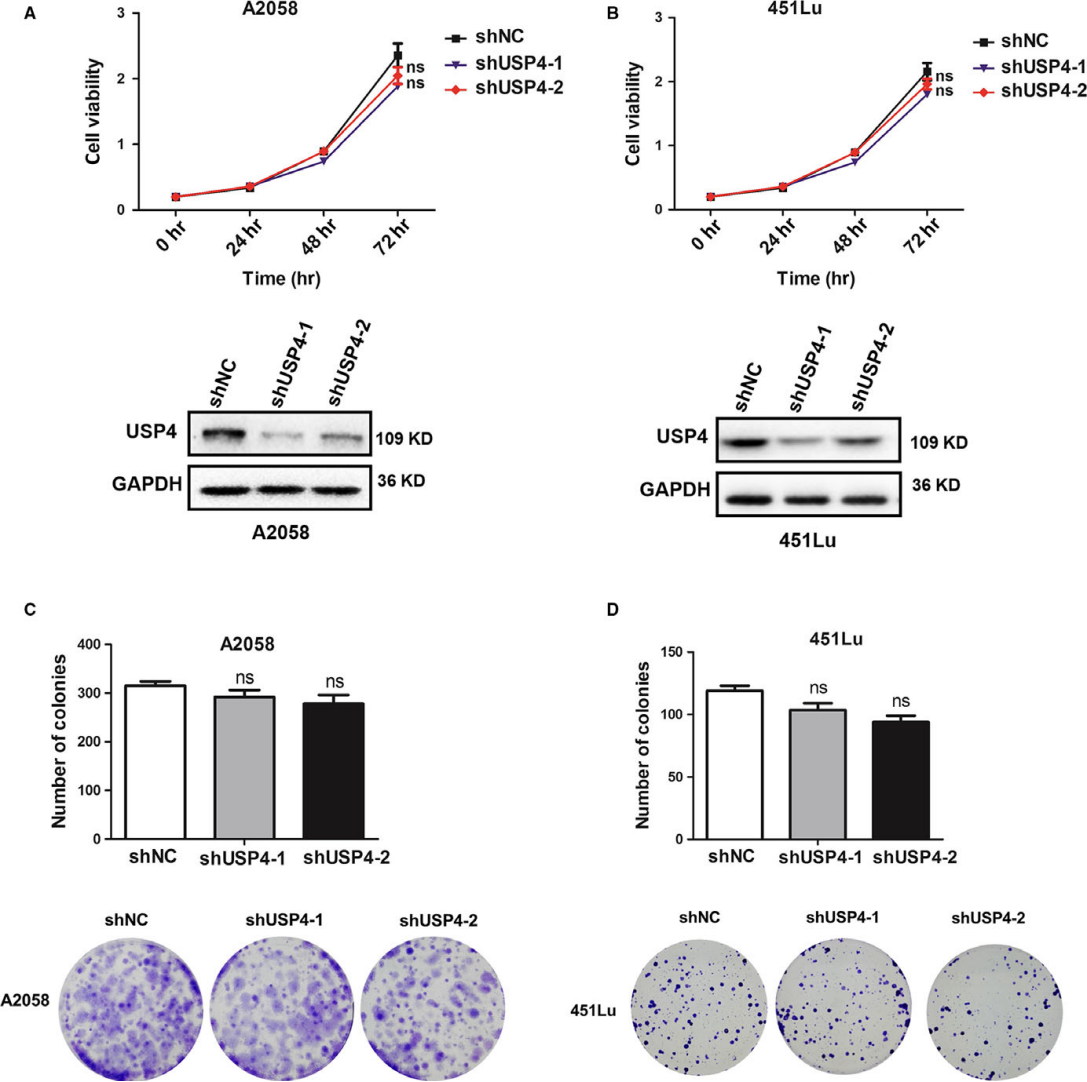
USP4 is dispensable for melanoma cell proliferation. A and B, Cell proliferation of A2058 and 451Lu cells was measured by CCK‐8 assay at 0, 24, 48 and 72 h. The lower panel is the knockdown efficiency of USP4 expression. C and D, Cell colony formation of A2058 and 451Lu cells was measured by colony formation assay. Data are presented as the mean ± SD, ns, non‐significant; Student's *t* test

Thereafter, to investigate the biological function of USP4 in melanoma, we employed lentiviral transfection to obtain stable knockdown of USP4 expression in both A2058 and 451Lu cell line, and the knockdown efficiency was confirmed by western blotting (Figure 2A). Our cell counting kit‐8 (CCK8) assay showed that the proliferative rate of melanoma cells was not altered after USP4 knockdown (Figure 2A,B). Concurrently, the colony formation assay also revealed that USP4 deficiency had little impact on the clonogenic ability of melanoma cells (Figure 2C,D). Therefore, although UPS4 expression was significantly up‐regulated in melanoma, it indeed exerted no prominent effect on melanoma cell proliferation.

### 3.3 USP4 protects melanoma cells from cisplatin‐induced apoptosis in a p53‐dependent manner

**Figure 3:**
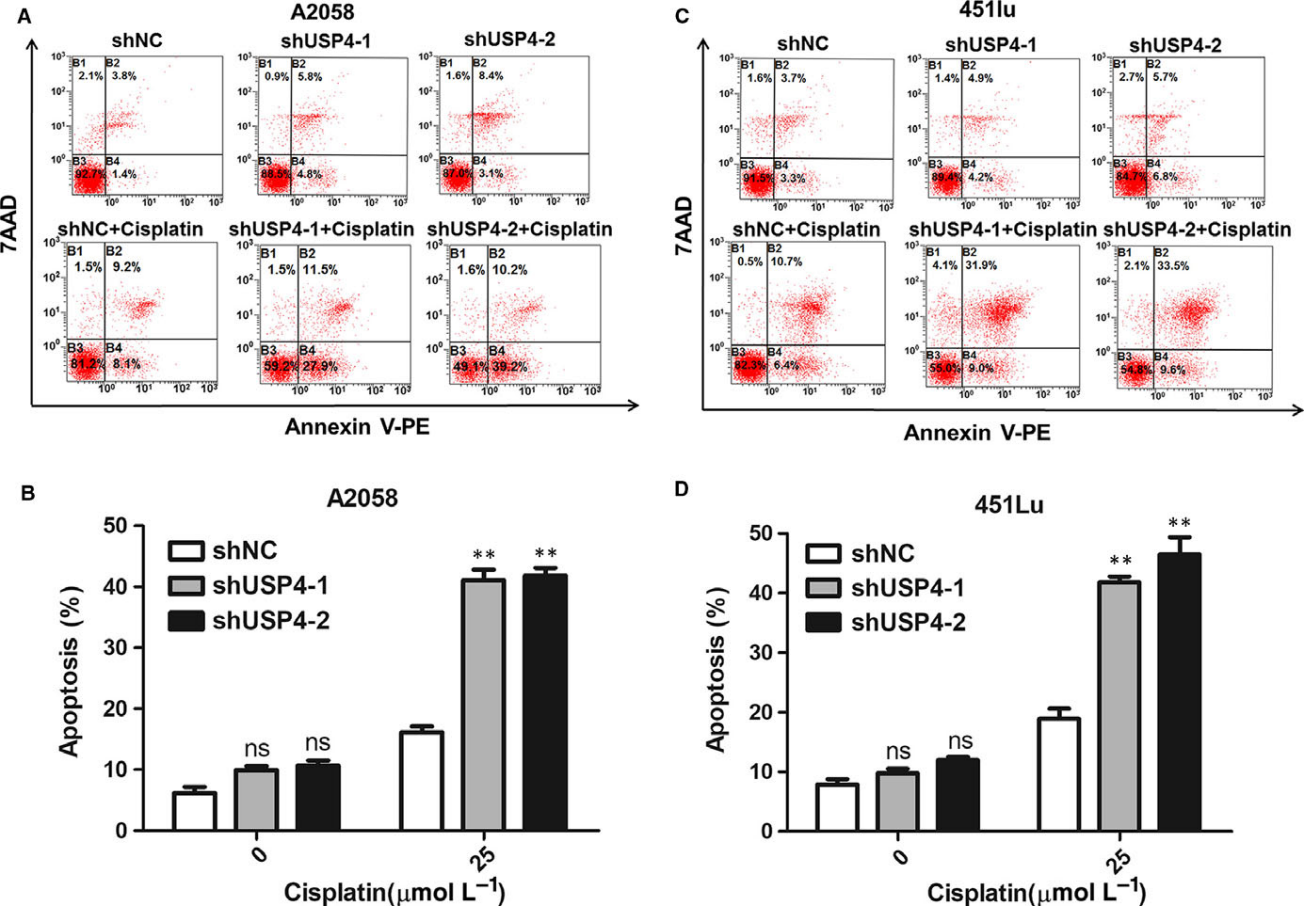
The knockdown of USP4 expression promotes melanoma cell apoptosis under cisplatin treatment. A and B, Representative flow cytometry images of cell apoptosis in A2058 cell with indicated treatment. The lower panel is the knockdown efficiency of USP4 expression. The lower panel is a densitometry analysis of 3 individual experiments. C and D, Representative flow cytometry images of cell apoptosis in 451Lu cell with indicated treatment. The lower panel is a densitometry analysis of 3 individual experiments. Data are presented as the mean ± SD, ns, non‐significant, *P <* *.05; Student's *t* test

**Figure 4:**
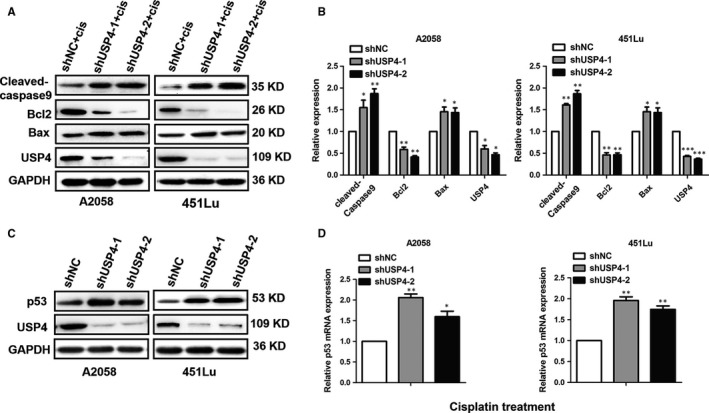
The knockdown of USP4 activates p53 signalling in melanoma cells under cisplatin treatment. A and B, The expressions of Cleaved‐caspase 9, Bcl‐2 and Bax was analysed by immunoblotting in melanoma cells with indicated treatment. The right panel is a densitometry analysis of 3 individual experiments. Cis represents cisplatin. C, The protein level of p53 was analysed by immunoblotting in melanoma cells with indicated treatment. D, The mRNA level of p53 was analysed by qRT‐PCR in melanoma cells with indicated treatment. Data are presented as the mean ± SD, *P <* *.05, **P <* *.01, ***P <* *.001; Student's *t* test

The escape from stress‐induced apoptosis is a critical hallmark of tumour cell. Cisplatin is a conventional chemotherapeutic agent for melanoma therapy by inducing DNA damage. However, the intrinsic pro‐survival signalling could abolish the killing efficiency and lead to cisplatin tolerance and tumour development of melanoma.^23^, ^24^ Since that USP4 played a critical role in DNA damage response,^16^, ^17^ we proposed that the intervention of USP4 may affect cisplatin‐induced cell apoptosis in melanoma. To this end, we performed flow cytometry analysis to determine the cell apoptotic rate of cisplatin‐treated melanoma cells. As a result, cisplatin treatment was able to induce prominent cell apoptosis in melanoma, which was consistent with previous report.^24^ More importantly, although knockdown of USP4 expression alone had little impact on cell apoptosis, it could significantly sensitize melanoma cells to cisplatin‐induced cell death (Figure 3), suggesting that USP4 was a critical pro‐survival molecular in response to cisplatin treatment. Furthermore, we performed immunoblotting analysis to examine the expression of apoptosis‐associated molecules, including cleaved‐caspase‐9, Bcl2 and Bax. Consistently, the expressions of pro‐apoptotic cleaved‐caspase‐9 and Bax were increased in cisplatin‐treated melanoma cells with USP4 deficiency, whereas the expression of anti‐apoptotic Bcl‐2 was deceased (Figure 4A,B). Taken together, these results showed that USP4 was able to protect melanoma cells from cisplatin‐induced apoptosis, thus inducing melanoma tolerance to cisplatin treatment.

Recently, it has been reported that USP4 could antagonize p53 signalling through its deubiquitinating activity.^15^, ^18^ Given that Bax and Bcl2 are 2 canonical downstream regulators of p53 in mediating cell apoptosis and were regulated by USP4 in response to cisplatin treatment, we speculated that pro‐survival effect of USP4 in response to cisplatin treatment may be associated with p53 signalling pathway. Through immunoblotting analysis, we found that the knockdown of USP4 led to a remarkable increase in p53 protein expression under cisplatin stimulation (Figure 4C). In consistent, our RT‐PCR analysis also showed that the mRNA level of p53 was significantly up‐regulated upon the knockdown of USP4 (Figure 4D). Therefore, USP4 deficiency may sensitize melanoma to cisplatin‐induced cell apoptosis in a p53‐dependent manner.

### 3.4 USP4 up‐regulation contributes to melanoma invasion and migration by promoting EMT

**Figure 5:**
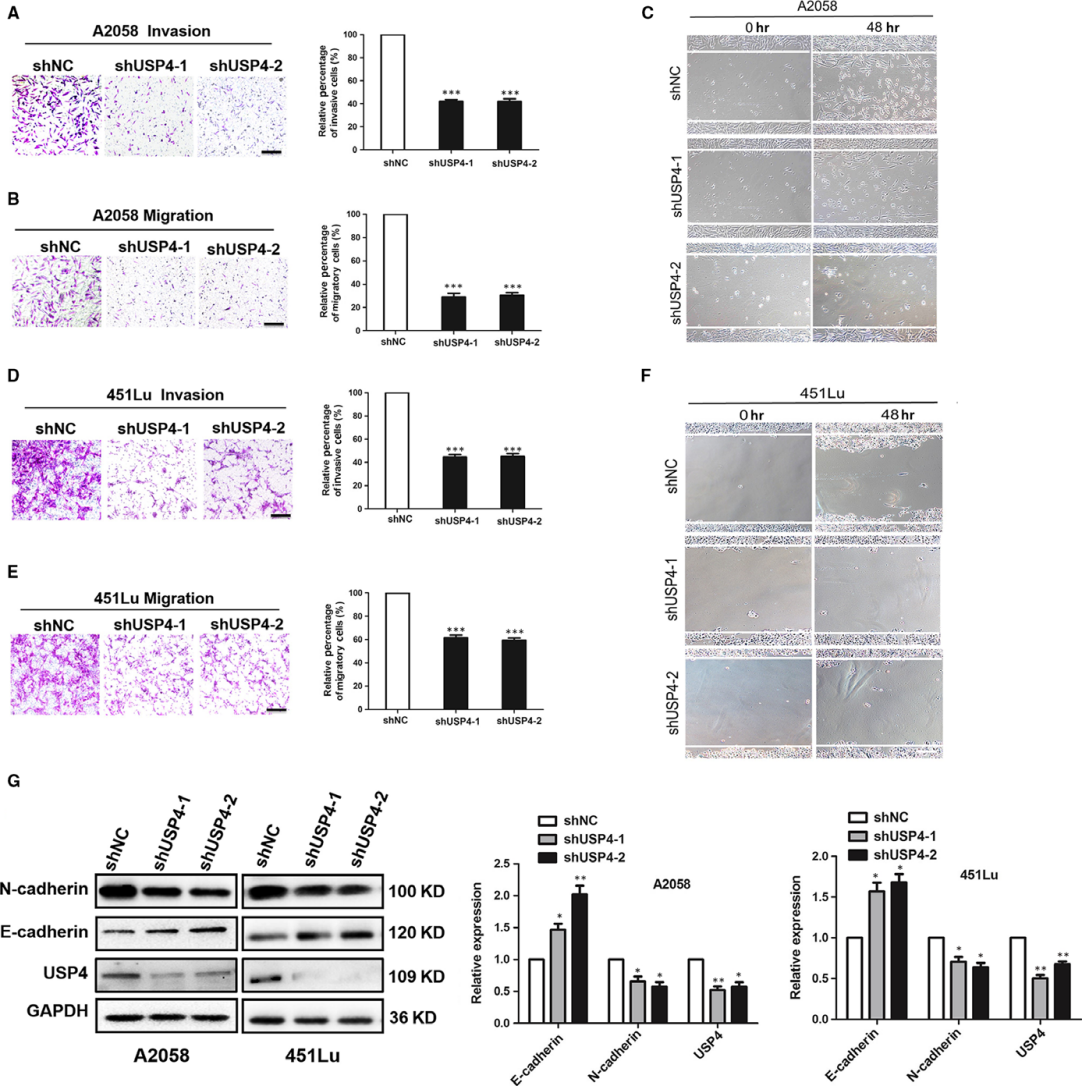
USP4 promotes invasion and migration of melanoma cell through the induction of EMT. A and B, A2058 cells with indicated treatment were subjected to the invasion and migration assay. Representative fields of invaded and migrated cells are shown. Scale bar = 100 μm. The invaded and migrated cells were also quantified on the right. Data represent the mean ± SD of triplicates. C, A2058 cells with indicated treatment were subjected to wound‐healing assay. Experiments were repeated 3 times with similar results. Scale bar = 100 μm. D and E, 451Lu cells with indicated treatment were subjected to the invasion and migration assay. Representative fields of invaded and migrated cells are shown. Scale bar = 100 μm. The invaded and migrated cells were also quantified on the right. Data represent the mean ± SD of triplicates. F, 451Lu cells with indicated treatment were subjected to wound‐healing assay. Experiments were repeated 3 times with similar results. Scale bar = 100 μm. G, Expressions of N‐cadherin and E‐cadherin were analysed by immunoblotting. The right panel is a densitometry analysis of 3 individual experiments. Data are presented as the mean ± SD, *P <* *.05, **P <* *.01; Student's *t* test

Since that the up‐regulation of USP4 expression was more prominent in metastatic melanoma, we further investigated whether USP4 could affect the migration and invasion of melanoma cells. Our transwell assay showed that the knockdown of USP4 remarkably reduced both the invasive and migratory capacity of melanoma cells (Figure 5A,B,D,E). In addition, the corresponding wound assays also revealed the delayed wound closure in melanoma cells with USP4 knockdown (Figure 5C,F), which was line with the observations in the transwell assay. Therefore, apart from the anti‐apoptotic effect in response to cisplatin, USP4 was also important in promoting the invasion and migration of melanoma cells.

The occurrence of tumour metastasis consists of a series of critical steps, among which the epithelial‐mesenchymal transition (EMT) is of great importance in enhancing tumour cell metastatic ability, and is generally characterized by the up‐regulation of N‐cadherin expression and meanwhile down‐regulation of E‐cadherin expression.^25^ Therefore, we turned to see whether USP4 exerted its impact on melanoma metastasis via EMT. Through the immunoblotting analysis, we found that the knockdown of USP4 induced drastic down‐regulation of N‐cadherin and up‐regulation of E‐cadherin (Figure 5G), indicating that USP4 deficiency could reverse the process of EMT. Moreover, we performed RT‐PCR experiment, and it also showed that the knockdown of USP4 could significantly decrease N‐cadherin and increase E‐cadherin expression at the transcriptional level (Figure 5G). Collectively, these data demonstrated that USP4 promotes melanoma cell migration and invasion by promoting EMT.

## 4 DISCUSSION

**Figure 6:**
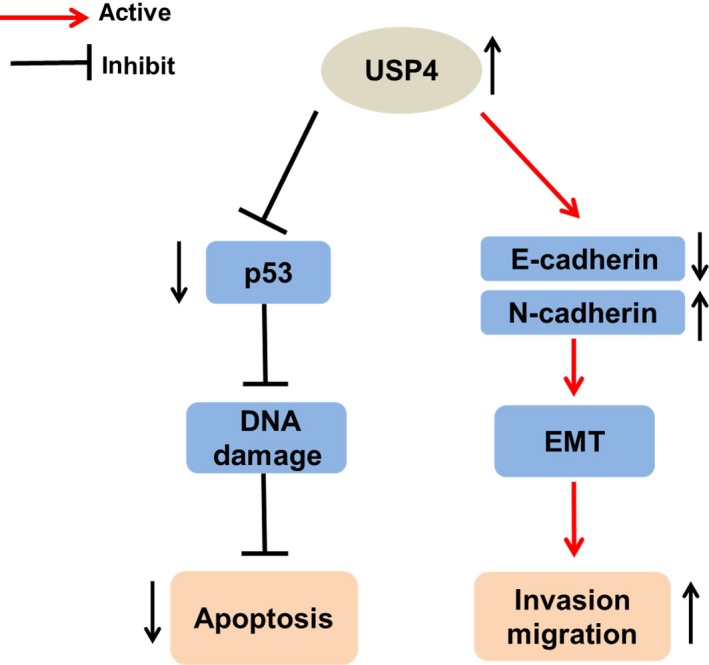
Proposed model of the oncogenic role of USP4 in melanoma. USP4 is significantly up‐regulated in melanoma. On the one hand, through the suppression of p53 signalling and DNA damage, USP4 is able to attenuate cell apoptotic rate in response to stress. On the other hand, though the induction of EMT, up‐regulated USP4 contributes to invasion and migration of melanoma cells

In the present study, we first found that the expression of USP4 was dramatically increased in both melanoma tissues and cell lines compared with benign nevus tissues and NHEM, respectively. In addition, USP4 expression in metastatic melanomas was significantly higher than that in primary ones. Our subsequent function study revealed that the intervention of USP4 expression had little impact on melanoma cell proliferation and colony formation. However, in response to cisplatin‐induced stress, the knockdown of USP4 could markedly increase the apoptotic rate of melanoma cells, and USP4 deficiency sensitized melanoma cell to cisplatin‐induced apoptosis by activating p53 pathway. Moreover, our results showed that USP4 could enhance the invasive and migratory capacity of melanoma cells through the induction of EMT. To sum up, we demonstrated that the up‐regulated USP4 plays an oncogenic role in melanoma by simultaneously suppressing stress‐induced cell apoptosis and facilitating tumour metastasis (Figure 6).

Ubiquitination is among the most evolutionarily conserved protein post‐translational modifications reversibly regulated by ubiquitin‐conjugating and ubiquitin‐deconjugating enzymes.^6^ Accumulative evidence has reported the diverse roles of ubiquitination in different biological activities and its great importance in the pathogenesis of various cancers, including melanoma.^11^ For example, the inactivating somatic mutations of *BAP1*, a nuclear ubiquitin carboxyl‐terminal hydrolase (UCH), were frequently detected in metastasizing uveal melanomas, and melanoma cell with *BAP1* gene deficiency grew as multi‐cellular non‐adherent spheroids with rounded epithelioid morphology, paralleled with increased metastatic capacity.^26^, ^27^ In addition, the mutation of ubiquitin ligase *FBXW7* resulted in hyper‐activation of Notch1 signalling and subsequent sustained proliferation of melanoma cells by up‐regulation of cell‐cycle mediators.^28^, ^29^, ^30^ What is more, the pro‐proliferative NF‐κB pathway was potentiated due to the facilitated degradation of IκB by E3 ubiquitin ligase β‐Trcp, which was up‐regulated by BRAF over‐activation in melanoma.^12^ Therefore, these results demonstrated the critical pathologic role of ubiquitination in melanoma development. However, previous studies mainly focused on the biological effect of ubiquitin‐conjugating enzymes, whether ubiquitin‐deconjugating enzymes were also greatly implicated in regulating melanoma progression remains far from understood. USPs represent the largest family of ubiquitin‐deconjugating enzymes. To date, the first well‐studied USP in melanoma was USP13, of which the significant up‐regulation contributed to melanoma growth via deubiquitination and stabilization of melanocyte lineage‐specific transcriptional factor MITF.^13^ Apart from this, USP8 and USP15 have been reported to act as oncogenic factor in melanoma, highlighting that both ubiquitin‐conjugating and ubiquitin‐deconjugating enzymes played important roles in melanoma tumour biology.^31^, ^32^ In the present study, we found that USP4 was another novel oncogene belonging to USP family with integrated effects on inhibiting cell apoptosis and facilitating tumour metastasis, further revealing the close linkage between dysregulated ubiquitination and cancer development. Hence, inhibition of USP4 could be not only a potential therapeutic approach for melanomas with high metastatic ability, but also a sensitizer for melanomas that are tolerant to conventional chemotherapeutic agent, especially cisplatin.

USP4 has been proved as a versatile regulator in terms of tumour biology. Specifically, up‐regulated USP4 potentiated the growth and invasion of colorectal cancer though deubiquitination and stabilization of PRL‐3.^19^ In addition, USP4 transduced Akt activation to TGF‐β signalling by deubiquitinating and stabilizing TGF‐β type I receptor, thus augmented breast cancer cell invasion and migration.^33^ These studies demonstrate USP4 as a powerful tumour promoter and an important determinant for canonical oncogenic signalling. However, in breast cancer, USP4 was also recognized as a tumour suppressor for its up‐regulatory effect on programmed cell death 4 (PDCD4) to restrain tumour growth.^20^ Moreover, USP4 was able to target TRAF2 and TRAF6 for deubiquitination and thereafter inhibited TNFα‐induced cancer cell migration.^34^ In the present study, we proved that USP4 expression was drastically increased in melanoma. More importantly, although USP4 was not essential for the proliferation of melanoma cells, it could exert its tumour‐facilitating role via the suppression of cell apoptosis and the promotion of invasive and migratory ability. Therefore, combined with previous studies, our results highlighted that the biological role of USP4 in cancer development is not only associated with tumour type, but also highly dependent on the context of malignant characteristics.

Recent 2 studies have identified USP4 as a critical regulator of DNA damage response through the interaction with CtIP and the MRE11‐RAD50‐NBS1 (MRN) complex to facilitate their recruitment to sites of DNA damage.^16^, ^17^ The endogenous USP4 could confer resistance to DNA damage agent by promoting DNA double‐stranded break (DSB) repair, resection and homologous recombination, whereas the knockdown of USP4 expression in U20S cancer cell significantly increased apoptotic cells in response to etoposide‐induced DNA damage.^15^ In line with these studies, our results also found that up‐regulated USP4 expression in melanoma mediated intrinsic tolerance to DNA damage agent cisplatin, further confirming the essential role of USP4 in DNA damage repair. More importantly, in response to cisplatin treatment, USP4 deficiency resulted in up‐regulation of p53 expression both at transcriptional level and translational level. Meanwhile, the pro‐apoptotic protein Bax was significantly increased, whereas the anti‐apoptotic protein Bcl2 decreased, both of which are canonical downstream genes of p53, indicating that the activation of p53 signalling was involved in the regulatory effect of USP4 on cell apoptosis. However, recent studies revealed that USP4 antagonized p53 activation by regulating its acetylation or ubiquitination modification without prominent influence on its mRNA level,^15^, ^18^ which is different from our results. The underlying mechanism needs to be further investigated.

USP4 has been greatly implicated in regulating tumour metastasis in breast cancer, lung cancer and colorectal cancer.^19^, ^33^, ^35^, ^36^ Notably, the loss of E‐cadherin expression accounted for USP4‐mediated augmentation of tumour metastatic capacity in all of these cancers. In line with previous studies, we also showed that USP4 promoted melanoma metastasis by suppressing E‐cadherin expression and increasing N‐cadherin expression, indicating the ubiquitous existence of the crosstalk between USP4 and EMT in different kinds of cancer. Importantly, Wnt signalling and TGF‐β signalling are 2 critical pro‐metastatic signalling pathways in various cancers with the positively regulatory impact on EMT, including melanoma.^37^ Several lines of evidence have revealed that USP4 was tightly associated with these 2 canonical pathways.^33^, ^36^, ^38^ Specifically, USP4 could directly interact with TGF‐β type I receptor (TRI) and stabilize TRI levels at the plasma membrane though its deubiquitinating activity, thereby maintaining the sustained activation of TGF‐β signalling.^33^ In addition, up‐regulated USP4 expression contributed to Wnt activation by interacting and stabilizing different components of Wnt signalling, including β‐catenin, Nemo like kinase (Nlk) and T‐cell factor 4 (TCF4).^36^, ^38^ Therefore, the facilitation of EMT and tumour metastasis by USP4 in melanoma may be also associated with the activation of TGF‐β and Wnt signalling, and this speculation needs to be further clarified.

In summary, our results demonstrate that USP4 is significantly increased in melanoma and plays an oncogenic role by simultaneously inhibiting stress‐induced cell apoptosis and promoting tumour metastasis. Targeting ubiquitination and ubiquitin‐related enzymes may be potential therapeutic approach for melanoma treatment in the future.

## CONFLICTS OF INTEREST

The authors state no conflicts of interest.
